# Supplementary material for: Comparing feedforward and recurrent neural network architectures with human behavior in artificial grammar learning
Source: Sci Rep. 2020 Dec 17;10:22172. doi: 10.1038/s41598-020-79127-y (PMC7747619; doi:10.1038/s41598-020-79127-y)
Supplement: Supplementary file 1 — Supplementary Information. [file 41598_2020_79127_MOESM1_ESM.docx]

**Supplementary materials of:**

Comparing Feedforward and Recurrent Neural Network Architectures with Human Behavior in Artificial Grammar Learning

Andrea Alamia^1^, Victor Gauducheau^1^, Dimitri Paisios^1,2^, Rufin VanRullen^1,3^

1. CerCo, CNRS, Toulouse, 31055 (France)
2. - Laboratoire Cognition, Langues, Langage, Ergonomie, CNRS, Université Toulouse (France)
3. ANITI, Université de Toulouse, Toulouse 31055 (France)

Correspondence to: [andrea.alamia@cnrs.fr](mailto:andrea.alamia@cnrs.fr)

**Appendix A**

**
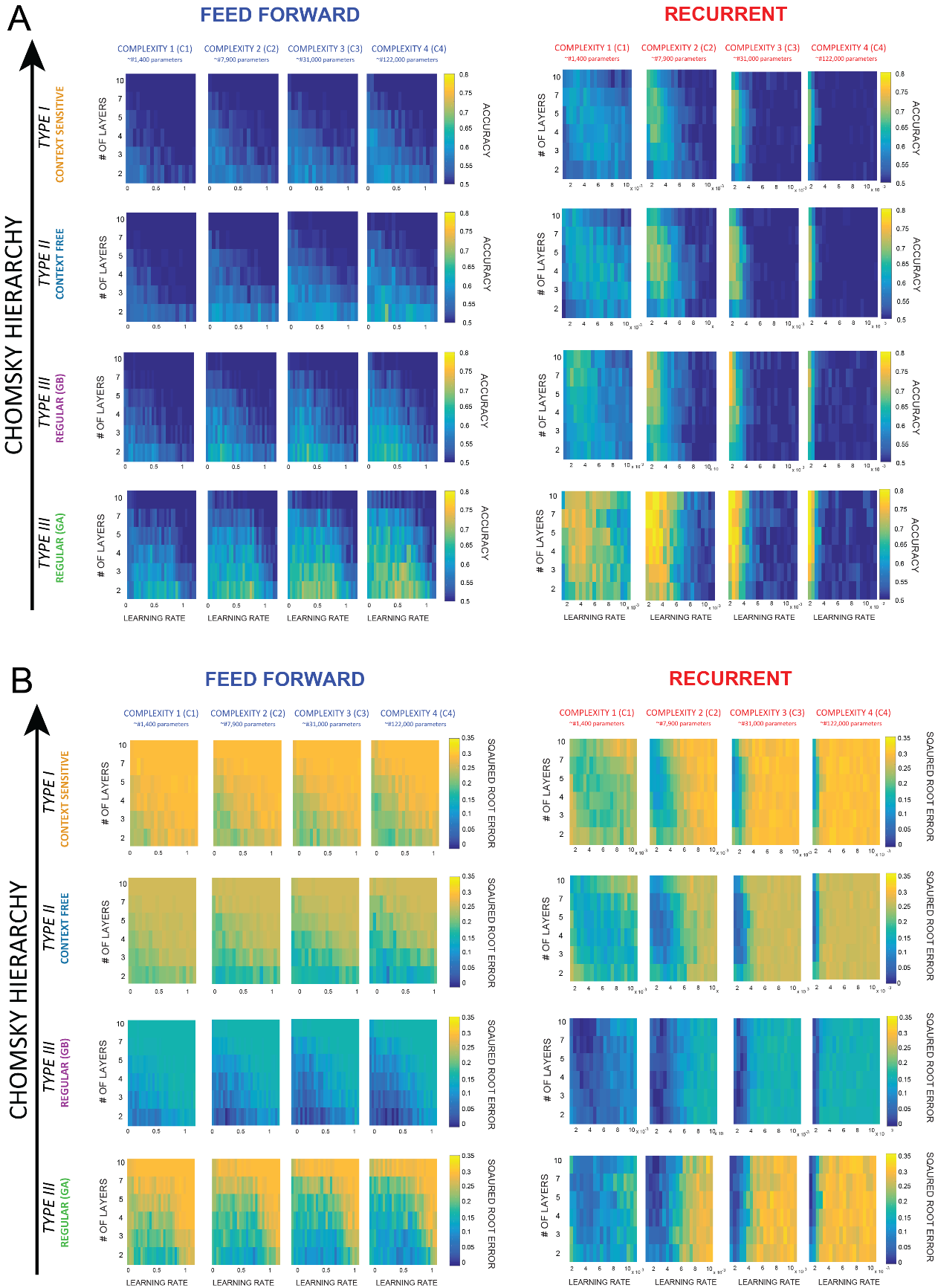
**

**Figure A.1**. Results of the parameter search for each complexity levels (columns, increasing number of parameters from left to right). Rows represent grammars ordered according to the Chomsky’s hierarchy. Subplot A shows the accuracy for feedforward (left) and recurrent (right) networks. Subplot B shows the squared root error, i.e. the distance to human performance. Non-surprisingly, the performance improves with more parameters, but similar patterns of results are observed between complexity levels: networks closer to human behavior have 2 layers in FF architectures, and lower learning rates in RR ones.

**
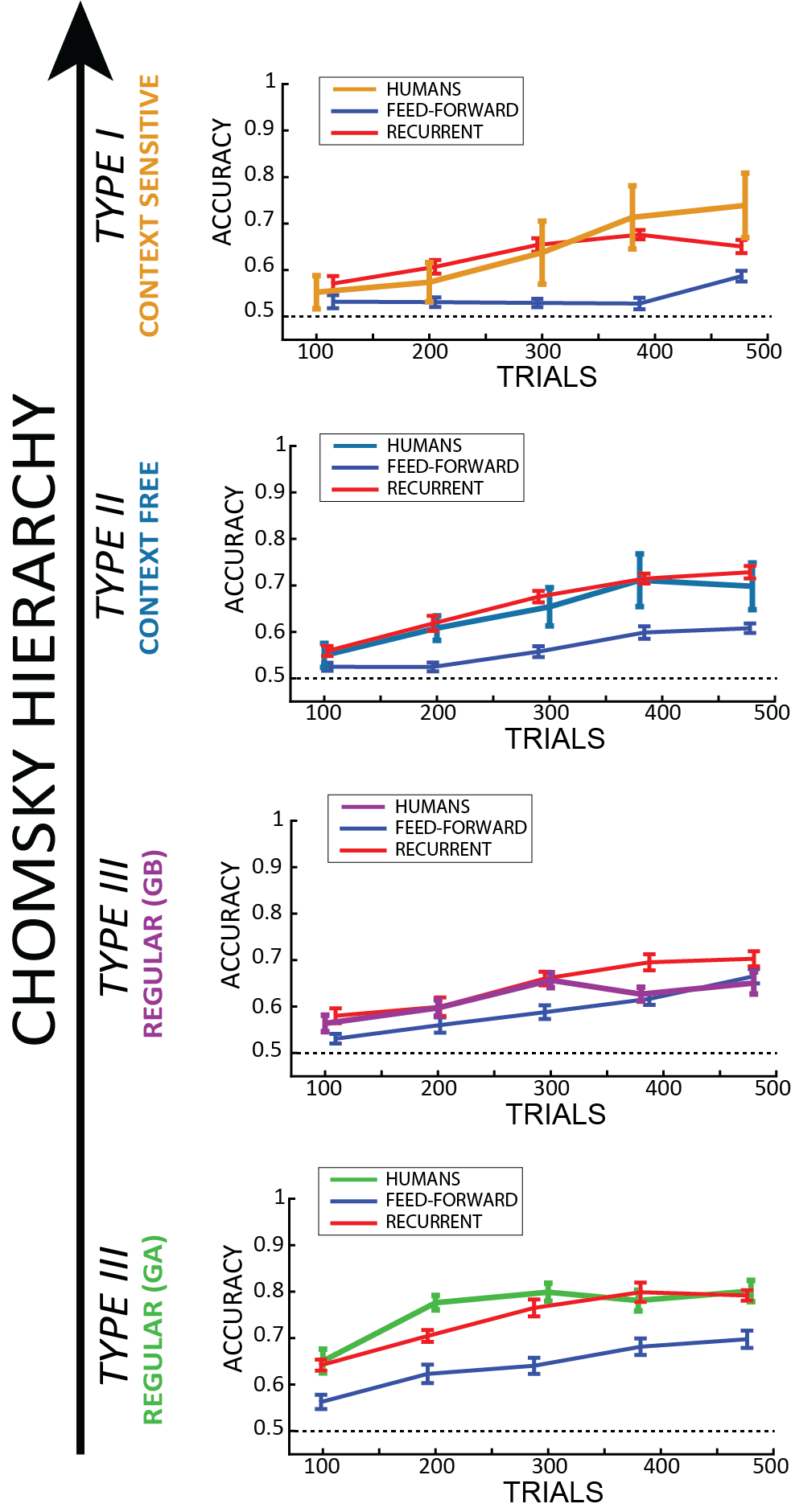
**

**Figure A.2**. The figure show the results of the FF and RR model which obtained the performance closest to human when averaging over all grammars (see figure 3 C,D). Notice that in each subplot, the FF and RR networks are the same across grammars. As shown in figure 4, the results confirm that RR architectures are closer to humans than FF, except in grammar B.

**
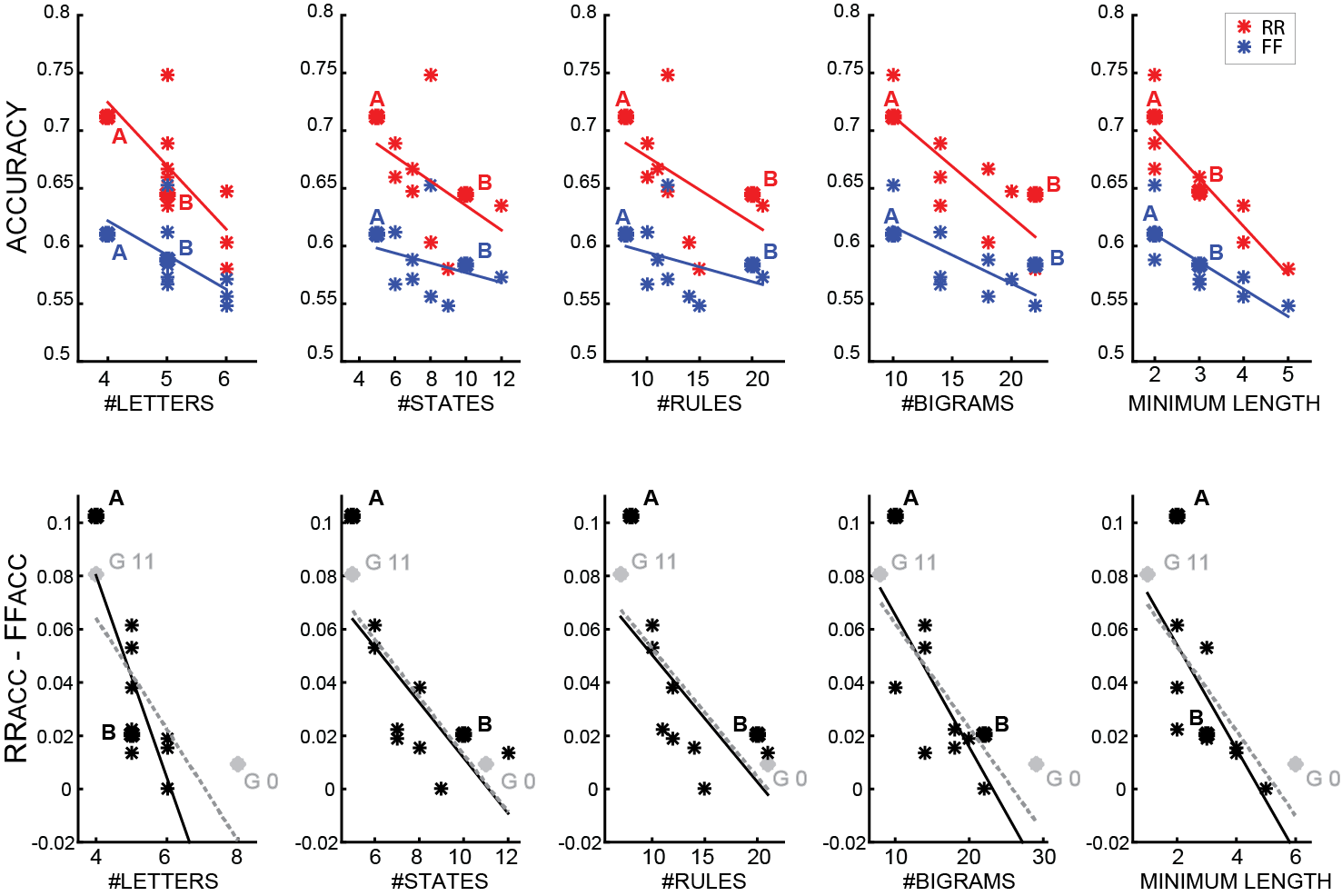
**

**Figure A.3**. Upper panel: correlation between the accuracy of FF (in blue) and RR (in red) networks with the different metrics used to evaluate grammar complexity. Grammar A and B are highlighted in each plot. Lower panel: same correlations but with the difference between RR and FF performance. Grammars A, B and grammars 0 and 11 are highlighted in each subplot.


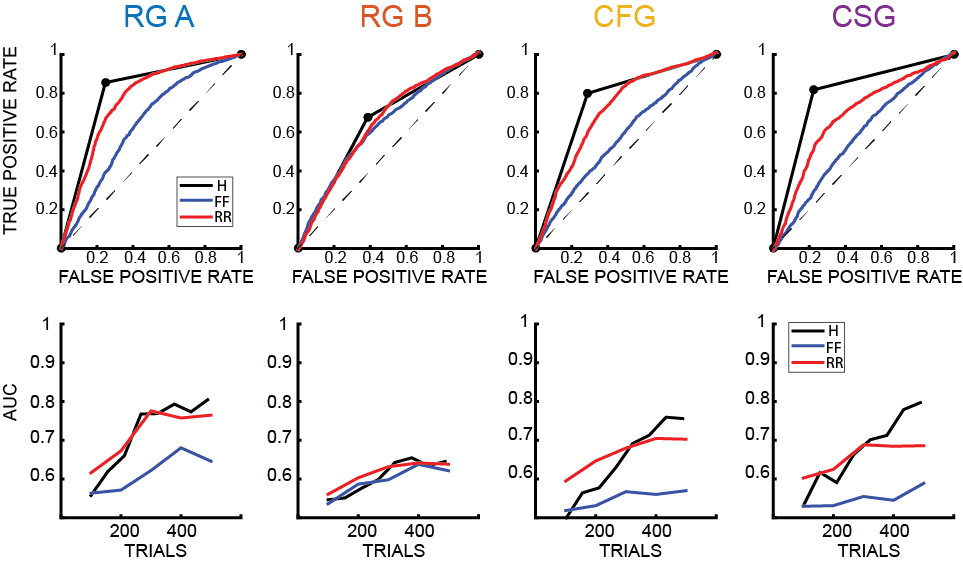


**Figure A.4**. – Results of the ROC analysis. The upper panels show the ROC curves computed for each grammar. For both feedforward (FF, in blue) and recurrent (RR, in red) the curve was computed after 500 trials of training, whereas we considered the data from the last block in the human experiment (H, in black). The lower panels show the Area Under the Curve (AUC) which quantifies the goodness of the classifier and it was computed as a function of trials. As in the main analysis of the manuscript, human and recurrent networks share a similar pattern of results, whereas feedforward network perform significantly worse (except for Grammar B) but above chance level.


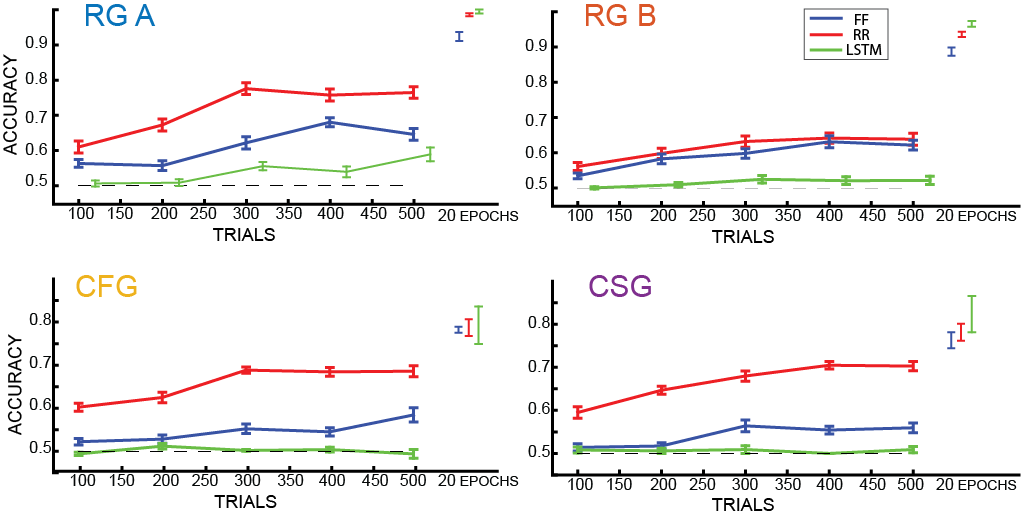


**Figure A.5**. – Comparing between FF, RR, and LSTM networks. Each plot shows the accuracy of feedforward (in blue), recurrent (red) and LSTM (in green) after one epoch, or after 20 epochs for each grammar. Except for RGA (top left plot), LSTM networks do not learn the rules of the grammars given such relatively little amount of trials; but they can reach comparable or better performance than the other models after 20 epochs of learning (see points on the right).

**Appendix B**

**Equations B.0**

$$accuracy= \frac{Number of correct responses}{Total number of responses}$$

$$sensitivity= \frac{True Positive}{True Positive+False Negative}$$

$$specificity= \frac{True Negative}{True Negative+False Positive}$$

**Table B.1 –** Results of the Bayesian ANOVA testing accuracy, comparing groups and the last three blocks for each grammar (rows). The factor AGENT is composed of FF, RR and Humans. The label ‘8’ refers to the last block of the implicit session, ‘M’ and ‘E’ refers respectively to the memory and explicit blocks. Each cell is composed of a BayesFactor and the percentage error in the form: BF(%err)**.**

| Grammars | AGENT | 8 vs E | 8 vs M | E vs M |
| --- | --- | --- | --- | --- |
| GA | 102.11 (0.009) | 889.7 (7e-7) | 1.2 (0.002) | 2.5 (8e-4) |
| GB | 1.22 e7 (7e7) | 1.1e6 (1e-10) | 0.4 (1e-4) | 7272.2 (9e-9) |
| CFG | 2.28 (0.008) | 3.96 (4e-4) | 1.6 (1e-5) | 0.35 (4e-5) |
| CSG | 7.44 (0.006) | 3.85 (1e-4) | 2.75 (1e-4) | 0.39 (0.005) |
| CFG (impl) | 18.37 (0.011) | 20.3 (5e-4) | 5.7 (4e-4) | 0.38 (0.005) |
| CSG (impl) | 10.11 (5e-4) | 4.71 (1e-5) | 3.5 (4e-4) | 0.4 (4e-6) |

**Table B.2 –** Results of the Bayesian ANOVA testing accuracy over AGENT (FF, RR and Humans, with post hoc comparisons), SEQUENCE LENGTH and their interaction. Each cell is composed of a BayesFactor and the percentage error in the form: BF(%err)**.**

| Grammars | AGENT | SEQ. LENGTH | INTERACTION | H vs FF | H vs RR | RR vs FF |
| --- | --- | --- | --- | --- | --- | --- |
| GA | 4.9e8 (0.8) | 1.2e56 (0.3) | 18.47(0.8%) | 4.5e7 (2e-14) | 0.26 (3e-5) | 6.9e7 (2e-13) |
| GB | 0.38 (0.022) | 6.5e26 (0.008) | 3.3e3(1.1%) | - | - | - |
| CFG (impl) | 1.2e5 (0.020) | 7.1e6 (3e-4) | 8.3e10(0.8%) | 3.2e3 (4e-7) | 0.21 (0.02) | 2.0e5 (1e-9) |
| CSG (impl) | 2.0e5 (0.010) | 1.2e8 (1e-4) | 5.8e10(1.2%) | 46 (1e-4) | 1.1 (0.004) | 4.8e5 (2e-8) |

| Complexity metrics: | Pearson’s r | Bayes Factor | Upper – Lower 95% CI |
| --- | --- | --- | --- |
| #LETTER | -0.796 | 20.834 | [-0.23 -0.93] |
| #RULES | -0.676 | 5.764 | [-0.10 -0.88] |
| #BIGRAM | -0.732 | 9.704 | [-0.15 -0.91] |
| #MIN.LEN | -0.678 | 5.823 | [-0.08 -0.87] |
| #STATE | -0.721 | 8.667 | [-0.14 -0.90] |

**Table B.3 –** Results of the Bayesian correlation between the FF-RR difference in performance and the complexity metrics in regular grammars. Further than the Pearson’s r index and the Bayes Factor, also 95% confidence intervals of the ‘r’ estimate are reported in the last column.

**Appendix C**

**Questionnaires C.1:** questionnaire provided to the participants after the regular grammar B, to test the level of rules’ awareness. A very similar questionnaire was provided after grammar A.

SbjNo : Gender : Age :

De-briefing questionnaire

*Please answer each question accordingly to the sequences you have been classifying during the experiment*

1. Which letter(s) is(are) more likely to be in the first position

M R T V X how confidence you feel about your response

0 (no confident at all) – 100 (very confident)

1. Which letter(s) is(are) more likely to be in the last position

M R T V X how confidence you feel about your response

0 (no confident at all) – 100 (very confident)

1. Which letter(s) is(are) more likely to be in the second position

M R T V X how confidence you feel about your response

0 (no confident at all) – 100 (very confident)

1. Which letter(s) cannot be presented twice consequently (e.g. in position 2 and 3, or in position 3 and 4, etc)

M R T V X how confidence you feel about your response 0 (no confident at all) – 100 (very confident)

1. Which letter(s) is(are) more likely to appear after the letter ‘X’

M R T V X how confidence you feel about your response

0 (no confident at all) – 100 (very confident)

1. Which letter(s) is(are) more likely to appear after the bigram ‘MX’

M R T V X how confidence you feel about your response

0 (no confident at all) – 100 (very confident)

1. Which letter(s) is(are) more likely to appear after the bigram ‘XT’

M R T V X how confidence you feel about your response 0 (no confident at all) – 100 (very confident)

**Questionnaires C.2:** questionnaire provided to the participants after the context-specific and context-free grammars to test the level of rules’ awareness.

SbjNo : Gender : Age :

De-briefing questionnaire

Please answer each question accordingly to the sequences you have been classifying during the experiment

1. Here a list of sequences that do not comply with the rules:
   - can you report (circle / underline) the error in each of them? (report your confidence about your response: from 0 to 100)
   - can you write the correct version of each sequence ? (report your confidence about your response: from 0 to 100)

| Sequence : | Correct sequence + confidence: |
| --- | --- |
| SEQUENCE I |  |
| SEQUENCE II |  |
| .. |  |
| SEQUENCE VII |  |
|  |  |
|  |  |
|  |  |
|  |  |
|  |  |

1. Which pattern(s) or rule(s) have you been following to perform the task?
